# Vitamin D Deficiency and Maternal Diseases as Risk Factors for the Development of Macrosomia in Newborns

**DOI:** 10.3390/children11101160

**Published:** 2024-09-25

**Authors:** Nazym Ailbayeva, Aliya Alimbayeva, Oxana Yurkovskaya, Raida Faizova, Sayat Tanatarov, Gulnara Taiorazova, Aizhan Abylgazinova, Andrey Orekhov, Ulzhan Jamedinova, Lyudmila Pivina

**Affiliations:** 1 Department of Pediatrics and Medical Rehabilitation Named after Tusupova D.M., Semey Medical University, Semey 071400, Kazakhstan; nazym.ailbayeva@smu.edu.kz (N.A.); aliya.alimbayeva@smu.edu.kz (A.A.); gulnara.taiorazova@smu.edu.kz (G.T.); aizhan.abylgazinova@smu.edu.kz (A.A.); 2 Department of General Medical Practice, Semey Medical University, Semey 071400, Kazakhstan; oksana.yurkovskaya@smu.edu.kz (O.Y.); raida.faizova@smu.edu.kz (R.F.); 3 Center for Nuclear Medicine and Oncology, Semey 071400, Kazakhstan; sayat.tanatarov@smu.edu.kz; 4 Department of Internal Medicine, Semey Medical University, Semey 071400, Kazakhstan; andrey.orekhov@smu.edu.kz; 5 Epidemiology and Biostatistics Department, Semey Medical University, Semey 071400, Kazakhstan; ulzhan.jamedinova@smu.edu.kz; 6 Department of Emergency Medicine, Semey Medical University, Semey 071400, Kazakhstan

**Keywords:** macrosomia, newborns, vitamin D, maternal obesity, maternal hypothyroidism, risk factors

## Abstract

*Background:* The aim is to assess the association between the level of vitamin D, maternal diseases, and macrosomia in newborns. *Methods*: This study included 258 full-term newborns (86 newborns with macrosomia; and 172 newborns with normal weight). Enzyme immunoassays for the determination of vitamin D were performed. *Results:* Newborns with macrosomia were statistically significantly more likely to have severe vitamin D deficiency compared with control (13.5 ± 6.7 ng/mL vs. 21.3 ± 12.1 ng/mL; *p* < 0.05). In the main group, severe deficiency was found in 40.7% of newborns, in the control group this rate was 5.8% (χ^2^ = 71,788, df = 3, *p* < 0.001). Multiple regression analysis shows that statistically significant risk factors for the development of macrosomia were vitamin D deficiency in the cord blood (OR = 2.29), maternal age over 36 years old (OR = 19.54), and hypothyroidism (OR = 9.35). *Conclusion:* the results of our study demonstrate relationship between macrosomia in newborns and vitamin D deficiency in the cord blood, maternal overweight and obesity, maternal age and thyroid disease.

## 1. Introduction

Birth weight over 4000 g, regardless of gestational age, is considered macrosomia [1]. The prevalence of macrosomia in both developed and developing countries is 3–15% [2,3]. In recent decades, this problem has become increasingly relevant for public health around the world. Macrosomia always increases the risk of trauma for both mother and child and can lead to various adverse perinatal outcomes [4,5]. The development of macrosomia is based on the state of maternal health.

Macrosomia and its adverse perinatal outcomes are associated with excessive weight gain during pregnancy by the mother [6,7]. During pregnancy, the need for macronutrients, calories, and supplementation of minerals and vitamins, namely vitamin D, increases, providing optimal quality and quantity of the diet for the mother and fetus [8].

Maternal vitamin D deficiency is common during pregnancy and is a public health problem worldwide [9,10,11]. Vitamin D is a fat-soluble substance and a prohormone [12]. Vitamin D promotes calcium absorption; it is involved in metabolism [13]. Vitamin D deficiency has long-term health consequences for the child [14]. During pregnancy, it is up to the mother to provide the fetus with the necessary levels of vitamin D to meet its increased growth and development needs [15]. Vitamin D plays a role in enhancing lipolysis and fatty acid oxidation while reducing adipogenesis [16]. In 2021, it was found that vitamin D deficiency in newborns is associated with obesity and metabolic diseases in adulthood [17]. Maternal vitamin D levels are a key modifiable risk factor for childhood obesity [18,19]. Wen J. et al. [20] showed that low maternal vitamin D levels were associated with macrosomia in newborns.

The relationship between newborn weight, their health status, and maternal nutrition is well known [21]. A study by Chinese scientists found a link between iron deficiency and iron deficiency anemia (IDA) in mothers and macrosomia in newborns (OR = 1.39) [22]. The results of this study are fully consistent with the data obtained by the Israeli authors [23].

This study aims to assess the association between the level of vitamin D, maternal diseases, and macrosomia in newborns.

## 2. Materials and Methods of Research

### 2.1. Characteristics of the Object of Study

The study was conducted in the Regional Perinatal Center, Semey City, the Republic of Kazakhstan, from January 2021 to December 2021. It included 258 newborns which were divided into 2 groups: the main group of newborns with macrosomia (body weight ≥ 4000 g, n = 86) and a control group of newborns with a normal birth weight (2500–4000 g, n = 172). The exclusion criteria were as follows: newborns with congenital malformations and genetic diseases, along with premature newborns. The sample calculations were performed using the Sample Size Calculator [24]. We used the number of births in the perinatal center per year (on average, 6000 births) as the population size and the percentage of newborns with macrosomia (on average, 6%) as the population proportion. With a confidence interval of 95% and a margin of error of 5%, the sample size was 86 newborns. We calculated the control group at a ratio of 1:2 (172 newborns with normal weight).

Enzyme immunoassays for the determination of vitamin D were performed for each newborn.

Information on the health status of mothers was collected, including their age, pre-pregnancy weight, pre-partum weight, thyroid disease status (hypothyroidism), and diabetes status. It was identified by copying data from documents for screening for pregnancy. The body mass index was calculated for each mother at the time of registration for pregnancy and before childbirth.

In mothers, IDA was identified by copying data from documents for screening for pregnancy, and in newborns, iron levels were determined by mass spectrometry of umbilical cord blood. In newborns, we studied the iron content in the umbilical cord blood immediately after birth.

### 2.2. Ethical Approval Details

This study was conducted in accordance with the Declaration of Helsinki and was approved by the Ethics Committee of Semey Medical University (Protocol No. 2 dated 10 November 2020).

All participants (mothers) signed a voluntary informed consent form. Mothers were informed about the processing of the data collection, with the subsequent publication of the results of the studies without specifying personal data.

### 2.3. Laboratory Tests

#### Determination of the Level of Vitamin D in Cord Blood

Laboratory studies included determining the level of vitamin D in umbilical cord blood by ELISA using the 25-OHD ELISA kit in all children under study. For quantitative determination of 25-OH Vitamin D (D2 and D3) in blood serum we used a 25-OH Vitamin D total ELISA kit (Demeditec Diagnostics GmbH, Kiel, Germany) and AIFR-01 UNIPLAN™ analyzer (No. 15166-11 manufacturer: CJSC “Picon”, Moscow, Russia).

Enzyme immunoassay for the quantitative measurement of total 25-OH-Vitamin D (25-OH-Vitamin D2 and 25-OH-Vitamin D3) in human serum as a physiological marker to aid the diagnosis of vitamin D sufficiency. The 25-OH-Vitamin D ELISA is a solid-phase enzyme-linked immunosorbent assay (ELISA) based on the principle of competitive binding and is measured on an absorbance reader.

The Tecan-IBL 25-OH-Vitamin D ELISA is correlated against the IDHPLC-MS/MS reference method (the method is recognized by the Joint Committee for Traceability in Laboratory Medicine (JCTLM) as a reference measurement procedure (RMPs) of a higher-order).

The correlation was performed on 40 serum samples (CDC phase I samples, single-donor sera) comparing the results of TECAN-IBL 25-OH-Vitamin D ELISA to reference concentrations assigned by CDC using IDHPLC-MS/MS [25,26].

Vitamin 25(OH)D content was assessed based on the criteria of the Endocrine Society clinical practice guideline [27]: a concentration of 30–80 ng/mL is considered a normal rate; 20–30 ng/mL was estimated as insufficiency; deficiency means 10–19 ng/mL level, and <10 ng/mL we estimated as severe deficiency.

### 2.4. Statistical Analysis

Statistical data processing was performed using SPSS version 20.0 software (IBM Corp., Armonk, NY, USA). Quantitative data were assessed for their compliance with the normal distribution. Normally distributed scores were described using the arithmetic mean (M) and standard deviation (SD). Categorical data were described using absolute values and percentages using descriptive statistics. To determine the influence of the factors that we studied on children in the perinatal period, and determine the risk of macrosomia, we conducted a statistical processing of the available data by calculating logistic regression with a 95% confidence interval and using an estimate of the odds ratio. Differences between the compared variables were considered significant at *p* < 0.05.

## 3. Results

Comparative characteristics of risk factors for the development of macrosomia in newborns are shown in Table 1. There were 43 (50%) male newborns in the main group and 80 (46.5%) in the control group. The average birth weight of the newborns in the main group was statistically significantly higher compared with the control group (*p* < 0.001). The same trend was observed about the average length of children in the studied groups (*p* < 0.001). The minimum length in the main group was 50 cm, while the maximum length was 62 cm. In the control group, the length of newborns ranged from 47 cm to 58 cm. In the main group, the average Apgar score both at the end of the first and at the end of the fifth minute after delivery was statistically significantly lower compared with the control group. It is noteworthy that in children with macrosomia, the Apgar score in the first minute corresponded to the state of hypoxia [28]. The mean age of mothers who gave birth to children with signs of macrosomia was also statistically significantly higher in comparison with the control group (*p* < 0.001). The gestational age in the newborns of the studied groups did not display any significant differences. In the newborns with macrosomia at birth, a statistically significantly lower glucose level was determined (*p* < 0.001), and hyperbilirubinemia was observed more often (*p* < 0.01). In mothers who gave birth to children with macrosomia, the average body mass index was statistically significantly higher compared with the control group (*p* < 0.001) (Table 1).

In the main group the proportion of mothers with overweight and varying degrees of obesity was significantly higher compared with the control group, where mothers with normal weight predominated (*p* < 0.001). Diabetes mellitus, IDA, and hypothyroidism were also significantly more common in mothers who gave birth to children with macrosomia (*p* < 0.001) (Table 2).

Table 3 presents the data on vitamin D levels in the studied groups. In the main group, the vitamin D level was within the range of deficiency, while in the control group, the rates were within the range of insufficiency, and the difference was statistically significant (t = 5.759, *p* < 0.05).

In the main group, 40.7 % had a severe deficiency of vitamin D level vs. 5.8 % in the control group; 36.0% of children had a deficiency of vitamin D level. Normal level was observed only in 5.8% of children in the main group. In the control group, 41.9% of children had a deficiency of vitamin D level (41.9%), and most children in this group had a normal level (44.2%). The differences between the groups were statistically significant (*p* < 0.001) (Table 4).

We analyzed the risk of neonatal macrosomia by considering risk factors using logistic regression with 95% confidence intervals and odds ratio calculations (Table 5). Statistically significant risk factors for the development of macrosomia were maternal age over 36 years old (OR = 9.1), IDA (OR = 10.343), diabetes mellitus type 1 (OR = 6.673), diabetes mellitus type 2 (OR = 14.298), gestational diabetes (OR = 6.863), and hypothyroidism (OR = 4.12). Both maternal overweight and obesity had a statistically significant effect on the development of macrosomia (OR = 4.822). Vitamin D cord blood level, as a proxy for maternal vitamin D deficiency during pregnancy, may be associated with macrosomia; the adjusted OR for this factor was 4.059.

The multiple regression analysis showed that the risk of developing macrosomia increased in women aged 26–35 years by 3.631 times, over 36 years of age by 19.539 times, in the presence of maternal hypothyroidism by 9.353 times, and vitamin D deficiency by 2.288 times (Table 6). The IDA in a pregnant woman does not have any relationship with macrosomia.

**Figure 1:**
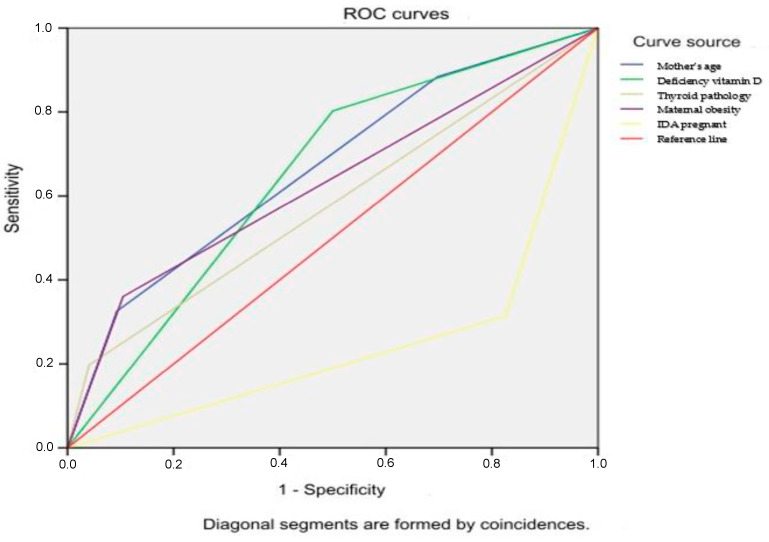
ROC curves characterizes the probability of developing macrosomia depending on the risk factors.

To assess the probability of developing macrosomia depending on the value of the OR obtained in the multivariate model, ROC analysis was performed with the construction of ROC curves. For the indicator of maternal age over 26 years, the area under the curve (AUC) was 0.665 ± 0.036 [95% CI 0.594; 0.736]; for vitamin D deficiency in cord blood, it was 0.651 ± 0.035 [95% CI 0.582; 0.720]; for hypothyroidism, the AUC was 0.578 ± 0.039 [95% CI 0.502; 0.655]; for maternal obesity, the AUC was 0.628 ± 0.039 [95% CI 0.552; 0.704]. For maternal IDA, the AUC was 0.244 ± 0.034 [95% CI 0.178; 0.311] (Figure 1).

## 4. Discussion

The results of our study show that the severity of vitamin D deficiency was more significant in newborns with macrosomia. Wen J et al. [20], in their study, also pointed out the high significance of vitamin D deficiency as a risk factor for macrosomia. The risk of having a baby with macrosomia has also been associated with a higher susceptibility to vitamin D deficiency in several other studies [29]. Walsh JM et al. [30] did not find any significant association between 25-(OH)D and maternal or fetal weight or obesity; however, they found an association between maternal 25-(OH)D levels and insulin resistance. The relationship between vitamin D concentrations in newborn umbilical cord blood and maternal blood has been observed in several studies. Thus, in a study by Ariyawatkul K, (2018) [31], a significant correlation between maternal and cord blood vitamin D levels was established (r = 0.86; *p* < 0.001). The same relationship between the content of vitamin D in the blood of newborns and their mothers was found by Brazilian researchers (r = 0.765; *p* < 0.001) [32]. These data are confirmed by the results of a systematic review published in 2016 [33].

The mechanism of macrosomia development in vitamin D deficiency requires a comprehensive study. One such mechanism may be the increased proliferation and differentiation of preadipocytes associated with impaired methylation of the VLDLR and HIF1A genes, leading to increased fetal weight [34]. A decrease in serum 25(OH)D concentration < 50 nmol/L was significantly associated with newly diagnosed obesity. In addition, vitamin D deficiency is associated with intestinal microbiota disturbances, increased fatty acid synthesis, impaired adipogenesis, secretion of adipocytokines (including leptin, resistin, and adiponectin), activated systemic inflammation, increased oxidative reactions in adipose tissue, and insulin resistance [35,36].

It should be noted that the opinions of the authors regarding the relationship of vitamin D deficiency with fetal growth and birth weight are contradictory. There is information in the literature on intrauterine growth retardation of the fetus in the third trimester in women with low concentrations of 25OHD in the blood [37]. A positive correlation has been established between the level of vitamin D in the mother’s blood and fetal weight [38]. These results can be explained by the development of placental inflammation in vitamin D deficiency, as well as insufficient intake of calcium, which is necessary for the mineralization of fetal bones [39].

Our results show that maternal BMI gain, maternal age, hypothyroidism, and IDA during pregnancy are the most important predictors of neonatal macrosomia. Similarly, Lewandowska M (2021) [40] and Owens L (2010) [41] identified maternal BMI as the main predictor of macrosomia. Increased BMI has also been considered by other authors as a risk factor for macrosomia [42,43,44]. Understanding the role of maternal overweight and obesity in macrosomia development confirms the need for the monitoring and timely correction of the nutritional status of women before and during pregnancy to reduce the occurrence of this adverse outcome in newborns.

The influence of maternal thyroid disease on neonatal macrosomia was confirmed by the results of Dutch scientists who conducted a similar study in 2017 [45]. There was an inverse relationship between maternal free T4 in early pregnancy and birth weight, with a stronger association in male newborns. Maternal subclinical hypothyroidism in early pregnancy (TSH > 2.5 mU/L) was associated with an increased chance of macrosomia in male neonates (OR 1.95; 95% CI 1.22–3.11).

In the main group, IDA was found to be more common in mothers who gave birth to children with macrosomia. Similar results regarding the impact of maternal iron deficiency on neonatal macrosomia were obtained by Shi G. et al. [46] in northwest China. In our study, the level of serum iron in the newborns was within the normal range in both groups, although about 70% of the mothers of the children in the main study group were diagnosed with IDA. This can be explained by the fact that even with severe IDA in the mother, iron deficiency in children rarely develops due to the high permeability of the fetoplacental barrier [47]. The multiple regression analysis does not show a causal relationship between maternal IDA and neonatal macrosomia. This fact requires further study of the role of IDA and maternal iron deficiency in the development of macrosomia in newborns in larger study groups.

We believe that the strength of our study lies in the examination of the relationship between the level of vitamin D in the umbilical cord blood and the development of macrosomia in newborns, as such studies are relatively rare worldwide due to the need to obtain informed consent from mothers in labor. The limitation of our study is the small sample size of newborns with severe vitamin D deficiency.

## 5. Conclusions

The results of our study demonstrate the relationship between macrosomia in newborns and vitamin D deficiency in the cord blood, maternal overweight and obesity, maternal age, and hypothyroidism. Future research should focus on conducting detailed studies based on the creation of a specialized register and screening studies in both mothers and newborns.

## Figures and Tables

**Table 1:** Comparative characteristics of risk factors for the development of macrosomia in newborns.

Rate	Main Group	Control	*p*
	Mean ± SD	Mean ± SD	
Weight (grams)	4361 (±301.7)	3302 (±395.4)	0.001
Length (centimeters)	54.4 (±2.44)	52.9 (±2.41)	0.001
Head circumference (centimeters)	36.5 (±1.10)	35.2 (±1.59)	0.001
Chest circumference (centimeters)	36.5 (±1.98)	34.4 (±2.34)	0.001
Apgar at the end of 1 min (scores)	7 (±1.49)	8 (±0.99)	0.001
Apgar at the end of the fifth minute (scores)	8 (±1.49)	9 (±1.0)	0.001
Mother’s age (years)	32.4 (±5.54)	28.4 (±5.28)	0.001
Childbirth parity (number)	4 (±1.53)	2 (±1.06)	0.001
Gestational age (weeks)	38.9 (±1.53)	38.9 (±1.49)	0.95
Glucose level (mmol/L)	3.2 (±1.16)	3.6 (±0.80)	0.001
Hemoglobin (g/L)	194 (±30.6)	187 (±13.5)	0.06
Bilirubin (μmol/L)	27.3 (±16.4)	21.9 (±14.2)	0.01
Reticulocytes (%)	10 (±4.40)	9 (±3.65)	0.114
BMI at the time of pregnancy (m/h^2^)	27.2 (±9.66)	22.5 (±4.26)	0.001
BMI before childbirth (m/h^2^)	31.1 (±9.71)	25.5 (±4.86)	0.001

**Table 2:** Comparative characteristics of maternal concomitant diseases in the studied groups.

Pathology	Main Group	Control	*p*
Maternal obesity:			
Overweight	7 (8.1%)	6 (3.5%)	<0.001
Obesity 1st degree	11 (12.8%)	8 (4.7%)	<0.001
Obesity 2 degrees	9 (10.5%)	4 (2.3%)	<0.001
Obesity 3 degrees	4 (4.7%)	0 (0.0%)	<0.001
Diabetes mellitus:			
T1DM	5 (5.8%)	0 (0.0%)	<0.001
T2DM	5 (5.8%)	1 (0.6%)	<0.001
Gestational diabetes	12 (14.0%)	5 (2.9%)	<0.001
Impaired glucose tolerance	7 (8.1%)	3 (1.7%)	<0.001
Maternal hypothyroidism	23 (26.7%)	17 (9.9%)	<0.001
Maternal IDA	59 (68.6%)	30 (17.4%)	<0.001

**Table 3:** Vitamin D levels in study groups (ng/mL).

Vitamin D Rate	Average Rate	95% CI	Median	Min	Max	Statistical Significance
Main group n = 86	13.2 ± 6.7	11.7–14.6	11.05	1.3	35.1	t = 5.759 *p* < 0.05
Control group n = 172	21.3 ± 12.1	19.5–23.1	18.8	0.5	44.2	

**Table 4:** Vitamin D levels in neonates born with macrosomia and neonates with normal weight.

Vitamin D Level	Main Group	Control	Statistical Significance
Severe deficiency	35 (40.7%)	10 (5.8%)	ꭓ^2^ = 71.788, df = 3, *p* < 0.001
Deficiency	31 (36.0%)	72 (41.9%)	
Insufficiency	15 (17.4%)	14 (8.1%)	
Normal rate	5 (5.8%)	76 (44.2%)	

**Table 5:** Logistic regression analysis of the association between macrosomia in newborns, vitamin D deficiency in the cord blood, and maternal diseases.

Variable	Adjusted OR	(95% CI)	SEE	*p*-Value
Diabetes mellitus type 1	6.673	1.669–26.682	1.218	0.007
Diabetes mellitus type 2	14.298	1.636–124.961	0.515	0.016
Gestational diabetes	6.863	2.316–20.328	0.655	<0.001
Maternal IDA	10.343	5.665–18.886	0.307	<0.001
BMI gain during pregnancy:				
Overweight and obesity	4.822	2.499–9.309	0.335	<0.001
Maternal hypothyroidism	4.12	1.994–5.516	0.370	<0.001
Mothers’ age:				
26–35 years old	2.4	1.124–5.124	0.237	0.024
Over 36 years old	9.1	3.647–22.692	0.507	<0.001
Vitamin D deficiency in the cord blood	4.059	2.207–7.463	0.311	<0.001

OR—odds ratio; CI = confidence interval; SEE = standard error, BMI = body mass index; IDA = iron deficiency anemia.

**Table 6:** Multiple regression analysis of the association of macrosomia with vitamin D deficiency and maternal diseases.

Variable	Adjusted OR	(95% CI)	*p*-Value
Maternal IDA	0.086	0.041–0.181	<0.001
Mothers’ age:			
26–35 years old	3.631	1.467–8.989	0.005
Over 36 years old	19.539	6.19–61.682	<0.001
Maternal hypothyroidism	9.353	2.863–30.569	<0.001
Vitamin D deficiency in the cord blood	2.288	1.06–4.943	0.035

OR—odds ratio, CI = confidence interval.

## Data Availability

The data necessary to reproduce the results presented here are not publicly accessible, as the participants’ informed consent did not include public data sharing, but are available from the first author upon reasonable request.
